# Evaluation of urinary inflammatory index in rapid screening of urinary tract infection

**DOI:** 10.1038/s41598-020-76352-3

**Published:** 2020-11-09

**Authors:** Wanjian Gu, Weizhou Huang, Jie Zhang, Shining Qian, Huiling Cao, Liang Ge

**Affiliations:** 1 Department of Clinical Laboratory, Affiliated Hospital of Nanjing University of Chinese Medicine, No.155, Hanzhong road, Nanjing, China; 2 Department of Urology Surgery, Affiliated Hospital of Nanjing University of Chinese Medicine, Nanjing, China

**Keywords:** Microbiology, Urology

## Abstract

The objective of this study was to assess the diagnosis value of urinary inflammatory index (UII) and systemic immune-inflammation index (SII) for UTI. Nine inflammatory indexes including neutrophil-to-lymphocyte ratio, platelet-to-lymphocyte ratio, SII and six UIIs were calculated for Receiver operating characteristic curve analysis to select which one is suitable for the screening of UTIs or distinguishing the types of bacteria. UII3, which calculated from leucocyte esterase (LE), nitrite, white blood cells and bacteria, was preferentially used as an indicator for the diagnosis of UTI when the threshold was set at 0.53. UII2 was more suitable for the distinction between groups when the cutoff is set to 0.94. Appropriate urinary inflammation index calculated by rapid urinalysis of urine dipstick and urine sediment can help us to predict urinary tract infection and bacterial type, and reduce the workload and costs of urine culture.

## Introduction

UTIs are an inflammatory reaction of urinary tract epithelium caused by a wide range of pathogens invasion, including Gram-negative and Gram-positive bacteria as well as fungi, usually accompanied by bacteriuria and pyuria, which affecting about 150 million people worldwide every year^1^. *Escherichia coli* is the most important pathogen causing UTI, and other pathogens include *Enterococcus*, *proteus*, *Klebsiella*, *Pseudomonas aeruginosa*, etc.; hospital acquired UTI pathogens include *Staphylococcus*, *Candida*, etc^2,3^. Urinary system diseases include asymptomatic bacteriuria, symptomatic UTI and UTI related septicemia. Bacteriuria ≥ 10^5^ CFU/ml is the most basic condition for laboratory diagnosis of UTI^4^. Other symptoms such as dysuria, increased frequency of urination, hematuria and backache are also strong evidence for diagnosis of UTI^5^.

Quantitative midstream urine culture is the gold standard for the diagnosis of UTI, and antimicrobial susceptibility testing is helpful to guide the clinical treatment. However, although the traditional method of urine culture is time-consuming, due to the lack of other reliable diagnostic indicators or powerful stimulus for antibiotic treatment^6^, about 70–80% of urine culture results are negative^7^. Clinicians often obtain urine cultures in patients without localizing urinary symptoms or positive culture results, will reflect asymptomatic bacteriuria rather than infection, resulting in the overdiagnosis and overtreatment are also well-recognized problems, then de-adoption of routine urine culture testing was called to action^8^.

To address the shortcomings of urine culture, several researchers are trying to find a rapid screening method that can reduce the necessity of urine culture, which will have a significant impact on the overall turnover time and laboratory economy^5,9–11^. Frequently used screening items such as microscopy analysis for WBCs and bacteria as well as dipstick testing for LE and nitrite in urine are fast but with low sensitivity^12^. Although the combination of positive test results is very sensitive, the usefulness of the dipstick test alone to rule in infection remains doubtful, even with high pre-test probabilities^13^. Therefore, rapid screening and accurate prediction of culture results are needed for clinicians shorten the diagnosis time, improve the efficiency of Microbial Laboratory, and win the treatment opportunity for patients as early as possible.

Although many mature detection methods such as MALDI-TOF mass spectrometry, Fluorescence in situ hybridization (FISH), etc. and emerging diagnostic platforms, such as biosensors, microfluidics, and la-on-a-chip technology, demonstrate the potential for expedited UTI diagnosis using enhanced screening^14^, however, they are not widely used in general laboratories. Our study planned to generate a new parameter, UII which was calculated by the rapid test results including urine dipstick and urine sediment analysis, for auxiliary diagnosis of UTI.

## Results

### Characteristics of positive urine culture

In total, 7279 of 17,053 total urine cultures (42.68%) between January 1, 2016, and Dec 31, 2019 produced growth of ≥ 10^5^ CFU/ml bacteria. The average age of male (2643, 36.31%) and female (4636, 63.69%) patients was 71.66 ± 16.60 and 66.17 ± 16.81, respectively (*P* < 0.05), and the higher infection rate is focused in the age of 50–95 (Supplementary Fig. 1). Of the 7279 cultures with bacterial growth, 5992 cultures (82.32%) presented only growth of one bacterium, 497 cultures (6.83%) presented growth of two or three bacteria, and 789 cultures (10.84%) presented growth of *Candida* species.

**Figure. 1:**
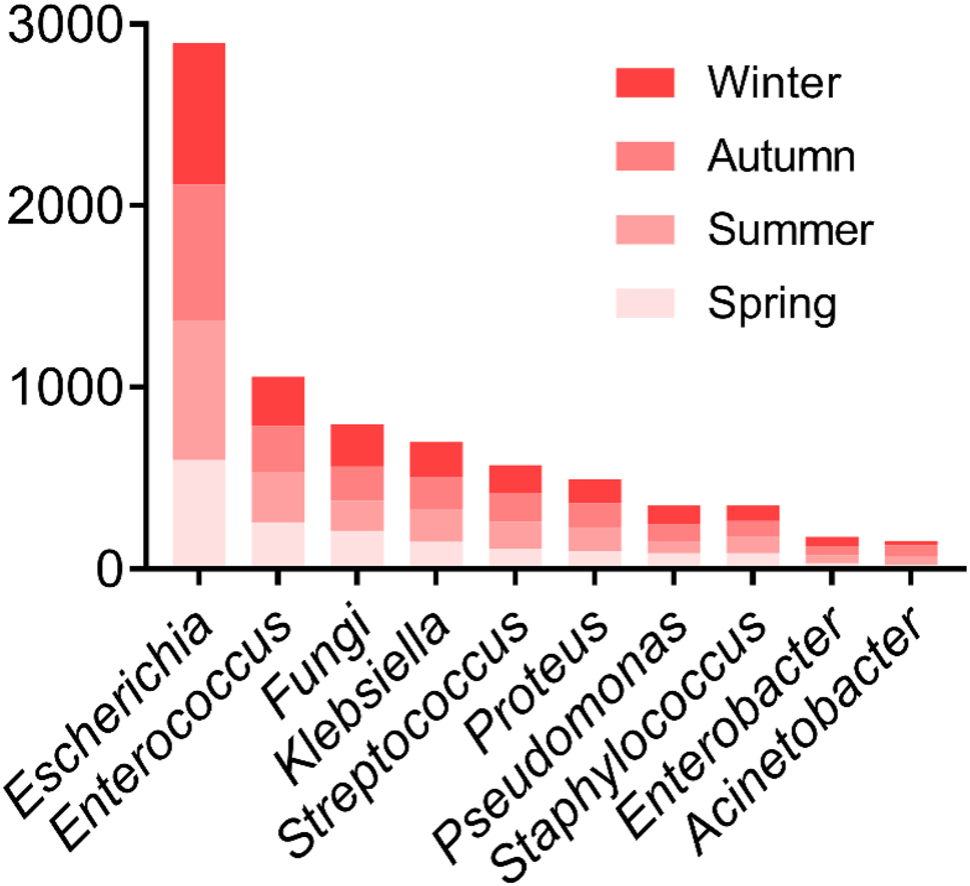
For the agents involved in the positive urine cultures, *Escherichia coli* is followed in prevalence by *Enterococcus*, *fungi*, *Klebsiella*, *Streptococcus*, *Proteus*, *Pseudomonas*, *Staphylococcus*, *Enterobacter* and *Acinetobacter* enter the top ten, and there was no significant difference in the infection rate among four seasons (*P* = 0.1033).

For the agents involved in the positive urine cultures, *Escherichia coli* is followed in prevalence by *Enterococcus*, *fungi*, *Klebsiella*, *Streptococcus*, *Proteus*, *Pseudomonas*, *Staphylococcus*, *Enterobacter* and *Acinetobacter* enter the top ten, and there was no significant difference in the infection rate among four seasons (Fig. 1). Then we analyzed the non-negative rate of each item of urinalysis among these bacteria, WBCs and LE were higher in each group, other indicators such as nitrite only had a higher positive rate in *Escherichia*, *Klebsiella*, *Pseudomonas* and *Enterobacter*, but almost no value for *fungi* and *Streptococcus*. This is in accordance with the characteristics of the reduction of nitrate to nitrite by *Enterobacteriaceae* (Table 1). The WBCs of *fungi*, *Klebsiella* and *Pseudomonas* were significantly higher than those of other genera (*P* < 0.001), there was no significant difference in WBCs between Gram-negative and Gram-positive bacteria (*P* = 0.6476) (Supplementary Fig. 2).

**Table 1:** Results of urinalysis as measured by the iRICELL3000.

Organism	No. of samples (missing value*)	WBCs median count/ul (range)	Non-negative rate (%)					
			WBCs	Leucocyte esterase	Nitrite	Bacterial	WBCC	Epithelial cells
*Escherichia*	589 (14)	185 (51–797)	492 (85.57%)	509 (88.52%)	370 (64.35%)	153 (26.61%)	286 (49.74%)	340 (59.13%)
*Enterococcus*	186 (9)	106 (16–470)	131 (74.01%)	129 (72.88%)	22 (12.43%)	28 (15.82%)	75 (42.37%)	48 (27.12%)
*Fungi*	112 (4)	464 (101–1880)	100 (92.59%)	94 (87.04%)	5 (4.63%)	8 (7.41%)	50 (46.30%)	28 (25.93%)
*Klebsiella*	121 (6)	370 (65–1806)	100 (86.96%)	101 (87.83%)	56 (48.70%)	25 (21.74%)	66 (57.39%)	30 (26.09%)
*Streptococcus*	134 (7)	68 (10–264)	86 (67.72%)	94 (74.02%)	7 (5.51%)	8 (6.30%)	37 (29.13%)	67 (52.76%)
*Proteus*	90 (9)	106 (12.5–596.5)	59 (72.84%)	63 (77.78%)	32 (39.51%)	5 (6.17%)	28 (34.57%)	27 (33.33%)
*Pseudomonas*	28 (2)	754.5 (180.75–1535.5)	25 (96.15%)	24 (92.31%)	15 (57.69%)	3 (11.54%)	20 (76.92%)	7 (26.92%)
*Staphylococcus*	55 (2)	69 (9.5–213)	39 (73.58%)	36 (67.92%)	12 (22.64%)	3 (5.66%)	16 (30.19%)	15 (28.30%)
*Enterobacter*	28 (2)	123.5 (21–693.5)	19 (73.08%)	24 (92.31%)	13 (50.00%)	2 (7.69%)	14 (53.85%)	7 (26.92%)
*Acinetobacter*	20 (1)	30 (6–261)	10 (52.63%)	12 (63.16%)	3 (15.79%)	0 (0.00%)	8 (42.11%)	4 (21.05%)
*Other bacterial*	64 (3)	72.5 (13–390.25)	42 (68.85%)	43 (70.49%)	20 (32.79%)	5 (8.20%)	23 (37.70%)	17 (27.87%)
*Negative control*	475 (4)	27 (6–124)	282 (59.87%)	256 (54.35%)	20 (4.25%)	23 (4.88%)	108 (22.93%)	118 (25.05%)
*Normal control*	459 (0)	4 (2–8)	23 (5.01%)	110 (23.97%)**	1 (0.22%)	3 (0.65%)	1 (0.22%)	126 (27.45%)**

*Missing value: The number of samples without urinalysis results.

**The number of leukocyte esterase and epithelial cell abnormalities in male urine was only 13 and 14 cases, respectively, while the female may have a high abnormal rate due to the influence of reproductive tract inflammation.

### Diagnostic performance of UII and SII for UTIs

**Figure. 2:**
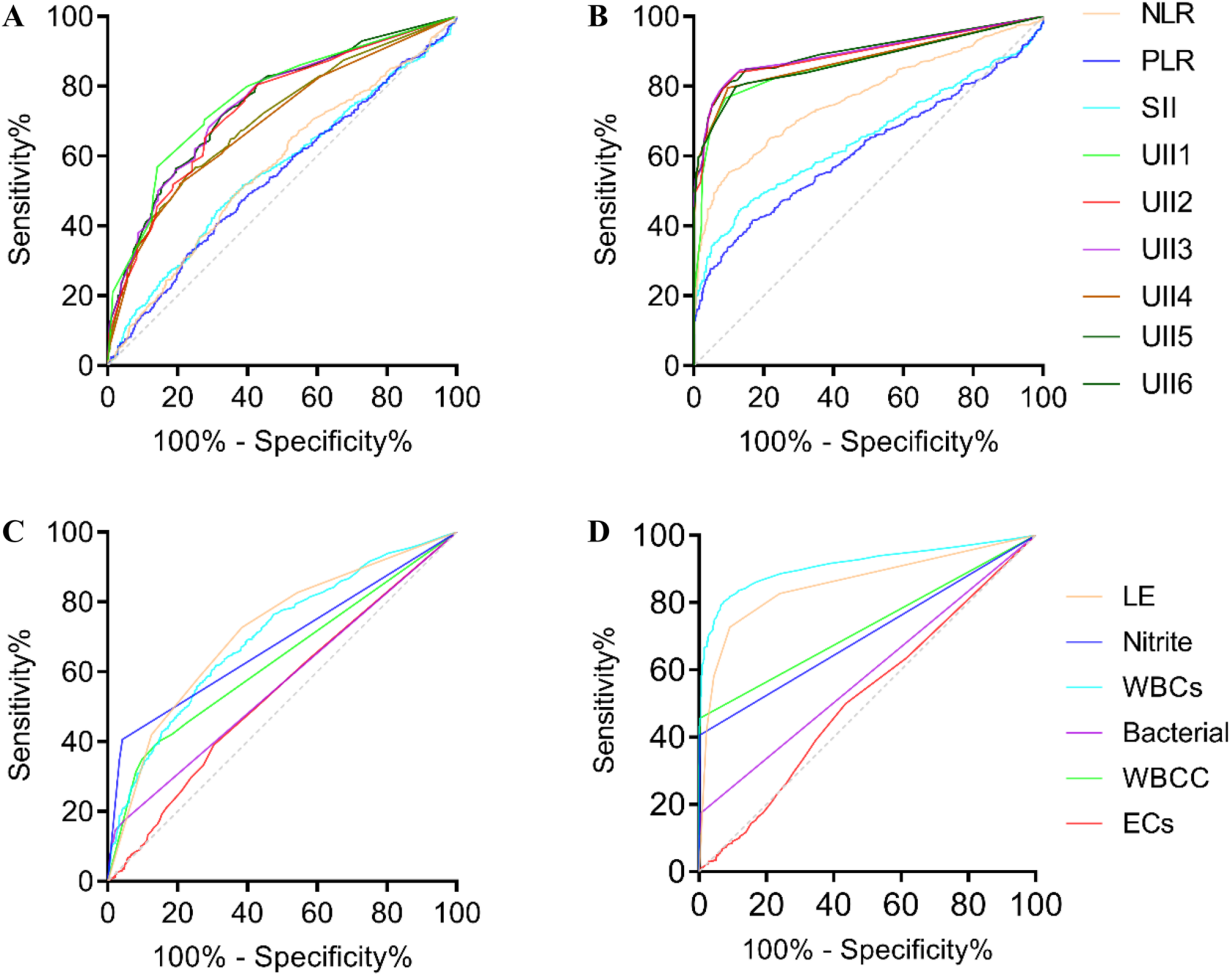
**A** and **B** ROC curves of NLR, PLR, SII and six UIIs compared with Negative controls and Normal controls, respectively. **C** and **D** ROC curves of LE, nitrite, WBCs, bacterial, WBCC and ECs compared with Negative controls and Normal controls, respectively.

The inflammation indexes for SII, NLR, PLR and different UIIs were calculated based on the data in 2019. The ROC curves and AUC for theses indexes are shown in Fig. 2A,B and Table 2. The diagnostic value of NLR, PLR and SII will not be considered in the following calculation due to their smaller AUC. Compared with the normal controls, the negative controls had lower AUC for UII1 to UII6. UII2 has the highest sensitivity of 88.4% at the cutoff of 0.44 while UII4 has the highest specificity of 99.3% at the cutoff of 0.14. UII3, with the highest AUC of 0.927 while the sensitivity and specificity were 84.3% and 95% at the cutoff of 0.53, become the preference diagnostic index for UTI screening. Meanwhile, the performance of individual items including LE, nitrite, WBCs, bacteria, WBCC and ECs were evaluated by the ROC curve. It is not difficult to see from Fig. 2C,D that LE and WBCs have higher AUC, keeping pace with the high non-negative rate in Table 1.

**Table 2:** Diagnostic performance of different inflammatory indexes.

Items	Compared with negative control					Compared with normal control				
	AUC	*P*	Cutoff	Sensitivity%	Specificity%	AUC	*P*	Cutoff	Sensitivity%	Specificity%
NLR	0.565	< 0.001	2.07	67.2	45.7	0.766	< 0.001	2.51	55.1	90.0
PLR	0.541	0.013	157.51	40.8	68.4	0.616	< 0.001	156.28	41.4	83.2
SII	0.556	0.001	608.60	44.0	69.1	0.648	< 0.001	600.02	44.5	86.7
UII1	0.774	< 0.001	0.75	70.5	72.2	0.886	< 0.001	0.53	79.9	90.8
UII2	0.744	< 0.001	0.74	80.4	57.1	0.924	< 0.001	0.44	88.2	90.8
UII3	0.752	< 0.001	1.14	68.1	71.1	0.927	< 0.001	0.53	84.3	95.0
UII4	0.698	< 0.001	1.44	52.3	79.0	0.916	< 0.001	0.18	83.6	99.3
UII5	0.706	< 0.001	1.43	57.1	74.7	0.882	< 0.001	0.53	73.1	91.9
UII6	0.748	< 0.001	0.95	71.4	66.2	0.913	< 0.001	0.48	84.5	86.9

**Figure. 3:**
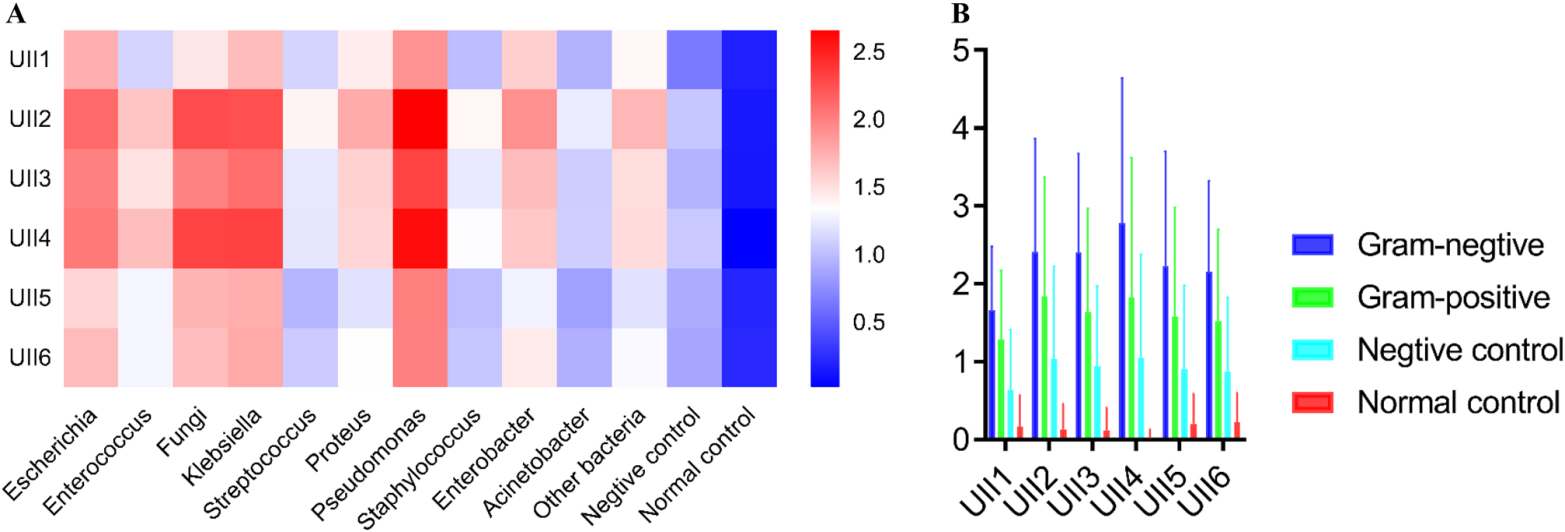
**A** Heatmap of six UIIs of different bacteria. **B** Comparison of six UIIs in different Gram staining.

The value of UII in the diagnosis of various bacteria is different. *Escherichia, fungi, Klebsiella, Pseudomonas and Enterobacter* have higher UII especially for UII2, UII3 and UII4, while *Streptococcus, Staphylococcus* and *Acinetobacter* have lower UII (Fig. 3A). In the same way, we found that the UIIs of Gram-negative bacteria were significantly higher than those of Gram-positive bacteria, and both of them were higher than those of the negative control and the normal control (Fig. 3B) (*P* < 0.001). Therefore, we may predict the bacterial type of UTI by UII values and clinical symptoms. Then, we chose UII2, UII3 and UII4 to distinguish the bacterial groups which group A contains *Escherichia, Enterococcus, fungi, Klebsiella, Pseudomonas and Enterobacter* while group B consists of *Streptococcus, Staphylococcus* and *Acinetobacter.* According to ROC curve analysis, AUCs of UII2, UII3 and UII4 were 0.623, 0.647 and 0.642 respectively (*P* < 0.001). Youden index analysis shows that when we set the cutoff of UII2, UII3 and UII4 at 0.94, 1.81 and 1.52 respectively, the sensitivity and specificity were 81.7% and 62.3% for UII2, 56.1% and 33.6% for UII3, 57.4% and 37.4% for UII4 (*P* < 0.001). Therefore, UII2 is more suitable for the distinction between groups when the cutoff is set to 0.94 (Supplementary Fig. 3).

## Discussion

It is generally believed that when UTI occurs, it is often accompanied by inflammation, and the number of red blood cells (RBCs), WBCs and bacteria in urine will increase. In addition to UTI, urinary WBCs may also come from female genital tract pollution, while some patients with low immunity may have normal urinary WBCs, and bacterial may also come from specimen contamination with periurethral, epidermal, perianal, and vaginal flora^15^, so the clinical diagnosis of UTI still mainly depends on the quantitative culture of bacteria in urine^16^. In addition, the discovery of the female urinary microbiota makes the sterile urine paradigm is no longer valid. Unfortunately, Culture-dependent techniques are severely limited because the vast majority of bacteria are not or cannot be cultured by standard clinical laboratory techniques^17^. Therefore, we hope to judge whether there is UTI by the results of routine urinalysis.

Rapid urine tests, such as microscopy, for bacteria and WBCs, and dipsticks, for LE and nitrite, are often used to guide early diagnosis and treatment of UTI. Many researchers have tried to compare the correlation between rapid urinalysis and positive urine culture. Generally, LE is more sensitive than the nitrite test in screening for UTI^18^ because of the high detection limit of the commercial dipstick. Urinary dipsticks are an effective rapid test for screening for asymptomatic, catheter-associated UTIs in intensive care unit patients^11^. However, traditional indicators such as LE and nitrite have high specificity but low sensitivity in the diagnosis of UTI, so their limited diagnostic value when used alone does not lead to satisfactory results. In this study, the non-negative rates of nitrite, WBCC, bacteria and ECs were all very low, which was not a powerful evidence for UTI screening, as reported by Koeijers et al.^19^. Another study reported the nitrite/nitrate ratio determined by the LC-MS/MS was significantly more sensitive (95%) and exhibited a satisfactory specificity (91%) in the screening of UIT^20^. Although WBCs and LE had high non-negative rates in various bacterial infections, they are not suitable for screening UTI alone. In order to improve the sensitivity and specificity of diagnosis, we combined the indicators related to inflammation in urinalysis to make a comprehensive judgment, so the UII came into being.

Multiple inflammation indexes such as NLR, PLR, SII and UII were calculated in this study for the screening of UTIs, only UII2, UII3, UII4 and UII6 with a higher AUC (> 0.9) while UII2 had the highest sensitivity of 88.2% and UII4 had the highest specificity of 99.3% at the cutoff of 0.44 and 0.18, respectively. NLR, PLR and SII are widely used in various tumors as useful prognostic indicators^21–23^. However, they seem to have no value in predicting UTIs, this suggests that UTIs are mostly focal inflammation, except for complicated UTI which is often accompanied by systemic diseases such as renal insufficiency, transplantation, diabetes or immune-deficiency^4^. LE, nitrite, WBCs and bacteria participate in the calculation of UII2, UII3, and UII4. This reminds us that these four indicators play an important role in UTI screening. Unfortunately, the AUC, sensitivity and specificity of each index decreased significantly when we compared to the negative control. Based on our data, only 18.7% (75/477) of patients collected 2 or 3 urine samples for culture, while 4.4% (21/477) of the patients with blood culture or sputum culture were positive, and 59.1% (282/477) of the patients with pyuria. Most of them did not take continuous specimens for testing, or may have been treated with antibiotics before specimens obtaining. Furthermore, a culture that shows significant bacterial growth may not reflect an active infection, for example, asymptomatic bacteriuria occurs in about 10% of community-dwelling older women^24^. Pyuria is a nonspecific finding that is frequent in older patients with or without bacteriuria^25^, while the negative predictive value can reach 95% or more to rule out infection if it is absent^26^.

The clinical treatment of UTIs mainly depend on experience and the adjustment of drug use according to the results of antimicrobial susceptibility test. With the spread of antibiotic resistance and its increasing threat to public health caused by UTIs became the second most common diagnosis for empirical antibiotics, national guidelines and antimicrobial management programs have been proposed to meet these challenges^27^. If we can predict the types of pathogens in advance, it will be of great value for the guidance of early experience drug use. In this study, UII was calculated by rapid urine test for the first time and compared with the results of urine culture. The AUC of 0.927 for prominent applications of UII3 which integrated the characteristics of four items and became the preferred prediction index according to Swets^28^. To our knowledge, the current study is the only report to focus on the value evaluation of UII in UTI screening.

UII can be used not only to judge the existence of UTI, but also to roughly identify the types of bacteria. In summary, prediction of UTIs by simple calculation of UII might be helpful in significantly reducing workload and costs of urine culture.

## Methods

### Urine culture and urinalysis

All patients are required to take midstream urine in sterile containers and submitted for bacterial culture. In the microbial laboratory, the uropathogens were isolated by using 1ul quantitative inoculation ring evenly smeared on the Columbia blood agar plate, cultured at 35 °C for 18–24 h, and continue to culture until 48 h when there is no bacterial growth. Then, they were identified by Vitek-2 compact Complete automated ID/AST platform and MALDI-TOF mass spectrometry (bioMérieux, France), growth of ≥ 10^5^ CFU/ml was considered to be a positive result of a urine culture^4^. In the instance of culture of > 1 microorganism, only the predominant microorganism was considered^19^.Urinalysis were performed for urine chemistry and microscopy values by the iRICELL3000 (IRIS diagnostic) automatic urinalysis line which were designed to streamline the UTI testing workflow by generating work lists of possible UTI patient sample values. The indicators measured by iChemVelocity were LE and nitrite, and the iQ200 module assessed were WBCs, white blood cell clot (WBCC), bacteria and epithelial cells (ECs).

### Blood cell analysis and SII

Serum concentrations of platelets, neutrophils, and lymphocytes were measured by the UniCel DxH Connected Workcell (Beckman diagnostic). SII, NLR, and PLR were calculated as follows: SII = platelet*neutrophil/lymphocyte, NLR = neutrophil/lymphocyte, PLR = platelet/lymphocyte^29^.

### Data collection and UII

A total of 7279 positive urine culture results were collected from the laboratory information system between January 1, 2016, and Dec 31, 2019. The 1597 results in total of blood cell analysis, urine dipstick and urine sediment in 2019 were collected simultaneously. Considering the changes of bacterial virulence, we gathered the blood cell analysis, urine dipstick and urine sediment results within 2 days before or after the urine culture^30^. According to the principle of randomization, we selected 500 outpatients or inpatients with negative urine culture results and 500 normal physical examination results as controls, those samples that are tested repeatedly or cultured to be positive again will be removed. Urine and venous blood were obtained and examined in the clinical laboratory Department of Affiliated hospital of Nanjing university of Traditional Chinese Medicine. Data collection was approved by the institutional review board and the Ethics Committee of the First Affiliated Hospital of Nanjing University of Traditional Chinese Medicine and all subjects included in the study gave their informed consent to the scientific use of the data. The methods were carried out in accordance with the relevant guidelines and regulations.

UII were calculated by the following formula: $UII = \sqrt {} \left( {\left( {a^{2}  + b^{2}  + c^{2}  + d^{2}  + e^{2}  + f^{2} } \right)/n} \right)$ where a, b, c, d, e, f are the converted values (Table 3) represent LE, nitrite, WBCs, bacteria, WBCC and ECs, n is the calculation items selected in the numerator. Different UIIs were calculated, such as UII_1_ for a and b, UII2 for a to c, UII3 for a to d, UII4 for c and d, UII5 for c to f, and UII6 for a to f, to decide which one is more suitable to predict UTI.

**Table 3:** Basic characteristics and result representation of each urinary item.

Item^a^	Attribute	Reference interval	Report format	Converted value^b^
Leucocyte esterase	Ordinal	Negative	Negative, ± , 1+, 2+, 3+, 4+	0, 0.5, 1, 2, 3, 4
Nitrite	Ordinal	Negative	Negative, 1+, 2+, 3+, 4+	0, 1, 2, 3, 4
WBCs	Quantitative	Male: 0–12/ul; Female: 0–26/ul	/ul	0, 1, 2, 3, 4, 5, 6, 7^c^
Bacterial	Ordinal	Negative	Negative, ± , 1+, 2+, 3+, 4+	0, 0.5, 1, 2, 3, 4
WBCC	Ordinal	Negative	Negative, occasionally, rare, few, medium, mass	0, 0.5, 1, 2, 3, 4
Epithelial cells	Quantitative	Male: 0–2/ul; Female: 0–5/ul	/ul	0, 1, 2, 3, 4, 5, 6, 7^c^

^a^ he results of leucocyte esterase and nitrite were obtained by urine dipstick analysis while other results were obtained by urine sediment test.

^b^To facilitate UII calculation, the report format of all parameters including quantitative and ordinal data are transformed to converted format. Ordinal data such as negative, ± , 1+, 2+, 3+, 4+ represent 0, 0.5, 1, 2, 3, 4, respectively. In addition, the ordinal data of white blood cell clot were converted in the same way.

^c^The rank data represent for within 1, 5, 10, 20, 30, 40, 50 and more than 50 times of the upper limit of the reference interval, respectively.

### Statistical analysis

Normally distributed continuous variables were compared by t-test, while non-normally distributed variables were compared by Mann–Whitney U test. Correlation of the non-negative rate of LE and WBCs was calculated by Pearson’s correlation analysis. ROC curve analyses were performed, and the AUC was used to assess the performance of UIIs and SII. All analyses were performed in SPSS software version 24.0 and GraphPad Prism version 8. *P* < 0.05 was considered statistically significant.

## Supplementary information

Supplementary Information
