# Supplementary material for: Evaluation of urinary inflammatory index in rapid screening of urinary tract infection
Source: Sci Rep. 2020 Nov 9;10:19306. doi: 10.1038/s41598-020-76352-3 (PMC7652836; doi:10.1038/s41598-020-76352-3)
Supplement: Supplementary file 1 — Supplementary Information [file 41598_2020_76352_MOESM1_ESM.docx]

## Evaluation of urinary inflammatory index in rapid screening of urinary tract infection

**Wanjian Gu^1,*^, Weizhou Huang^2^, Jie Zhang^1^, Shining Qian^1^, Huiling Cao^1^, Liang Ge^1^**

^1^Department of Clinical Laboratory; ^2^Department of Urology Surgery, Affiliated Hospital of Nanjing University of Chinese Medicine, Nanjing, China

* Corresponding author. Department of clinical laboratory, Affiliated Hospital of Nanjing University of Chinese Medicine, No.155, Hanzhong road, Nanjing, China. Tel. +86 025 86615175.

E-mail address: arrowsgu@163.com (Wanjian Gu)

ORCID: 0000-0003-3781-4668 (Wanjian Gu)

| 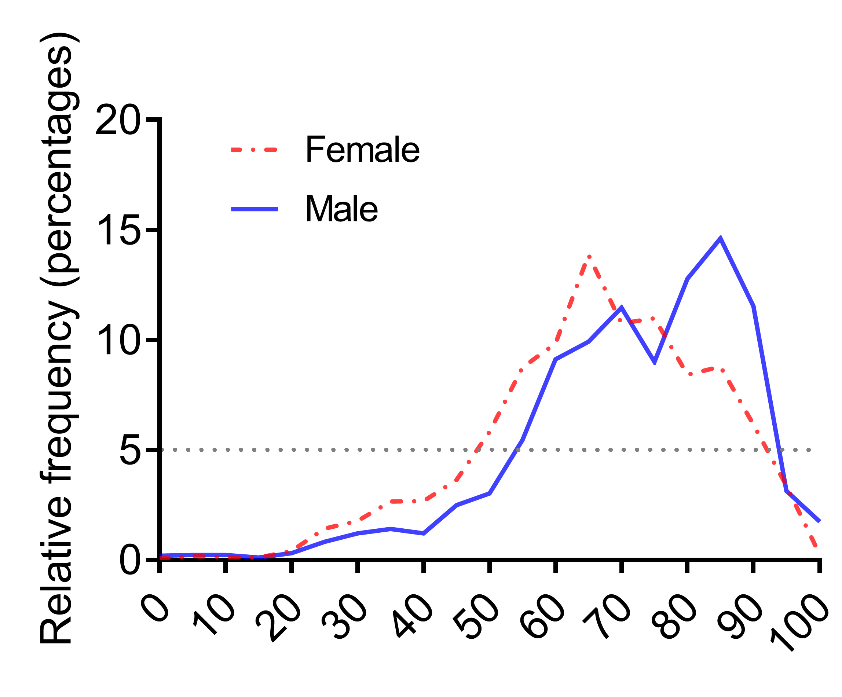 |
| --- |
| **Supplementary Figure 1 -** Characteristics of relative infection rate for female and male. The age with higher infection rate in both men and women is between 50 and 95 years old, as shown in the figure, the part above the gray dotted line is the age group with infection rate more than 5%. |

| **A** | 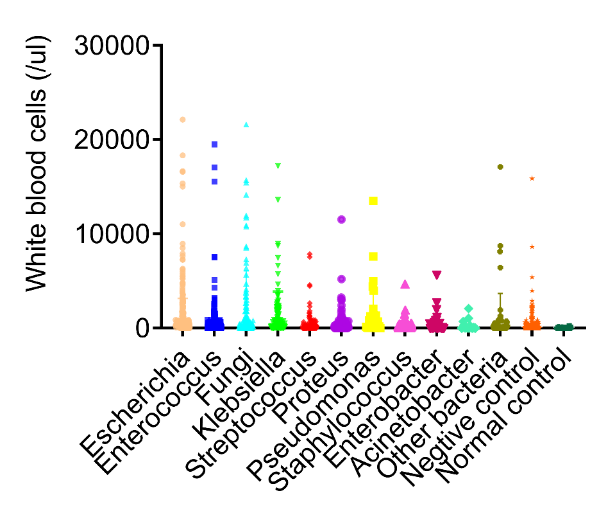 | **B** | 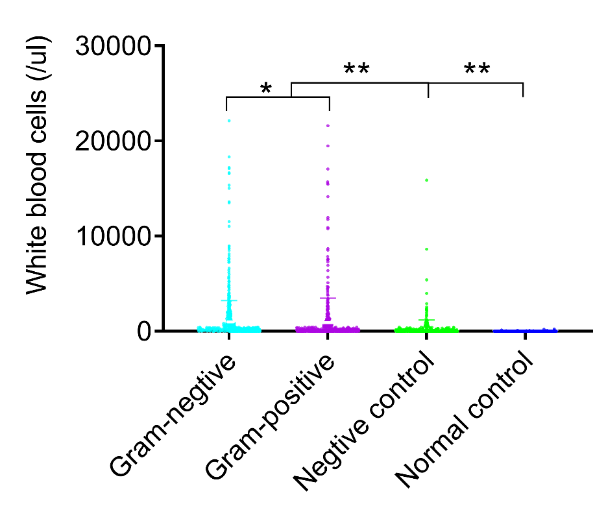 |
| --- | --- | --- | --- |
| **Supplementary Figure 2 -** A: The leukocytes of *fungi*, *Klebsiella* and *Pseudomonas* were significantly higher than those of other genera (P<0.001). B: There was no significant difference in WBCs between Gram-negative and Gram-positive bacteria (*P=0.6476), but they were significantly higher than the negative control and the normal control (**P<0.001). | | | |

| 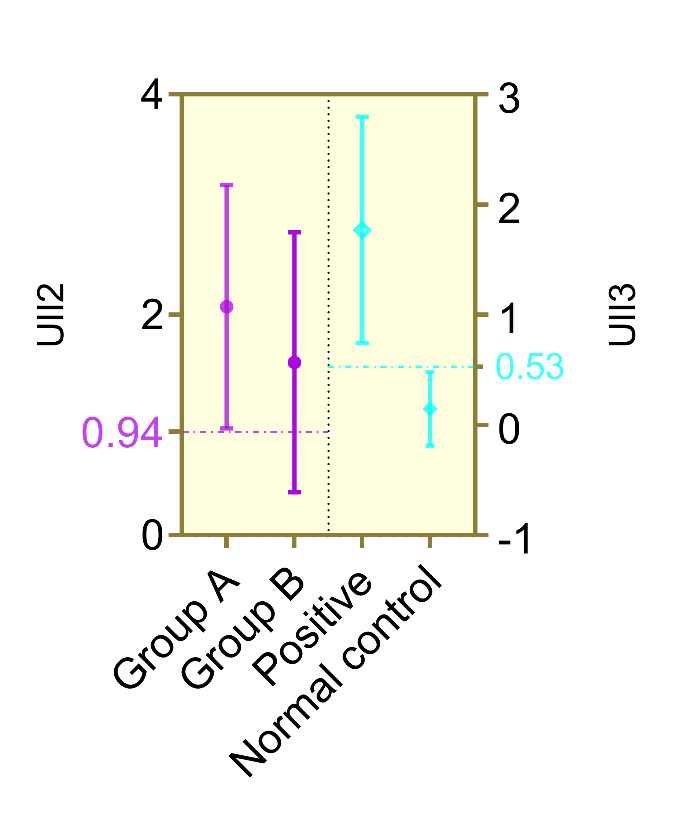 |
| --- |
| **Supplementary Figure 3 -** In the left part, UII2 is used for differential diagnosis of group A and B bacteria at the threshold of 0.94; for the right part, UII3 is used to judge whether there is UTI at the threshold of 0.53. |
